# Fat Embolism Syndrome: A Case Report and Review Literature

**DOI:** 10.1155/2018/1479850

**Published:** 2018-04-29

**Authors:** Nattaphol Uransilp, Sombat Muengtaweepongsa, Nuttawut Chanalithichai, Nattapol Tammachote

**Affiliations:** ^1^Fellowship in Cerebrovascular Disease and Neurovascular Ultrasound, Faculty of Medicine, Thammasat University, Pathum Thani, Thailand; ^2^Department of Internal Medicine, Faculty of Medicine, Thammasat University, Pathum Thani, Thailand; ^3^Department of Orthopedic Surgery, Faculty of Medicine, Thammasat University, Pathum Thani, Thailand

## Abstract

Fat embolism syndrome (FES) is a life-threatening complication in patients with orthopedic trauma, especially long bone fractures. The diagnosis of fat embolism is made by clinical features alone with no specific laboratory findings. FES has no specific treatment and requires supportive care, although it can be prevented by early fixation of bone fractures. Here, we report a case of FES in a patient with right femoral neck fracture, which was diagnosed initially by Gurd's criteria and subsequently confirmed by typical appearances on magnetic resonance imaging (MRI) of the brain. The patient received supportive management and a short course of intravenous methylprednisolone.

## 1. Introduction

Fat embolism was first described by Zenker [1] in 1861 in a railroad worker with a thoracolumbar crush injury. In 1873, Ernst von Bergmann was first to make a clinical diagnosis of fat embolism in a patient who fell off a roof and sustained a comminuted fracture of the distal femur.

FES usually occurs within 48 hours after trauma or during surgical procedures in most patients; however, most patients are usually asymptomatic. A small number of patients may exhibit signs and symptoms involving the lungs, brain, and skin. The actual incidence of FES ranges from <1% to 30% due to a lack of universal criteria for diagnosis.

## 2. Case Presentation

**Figure 1:**
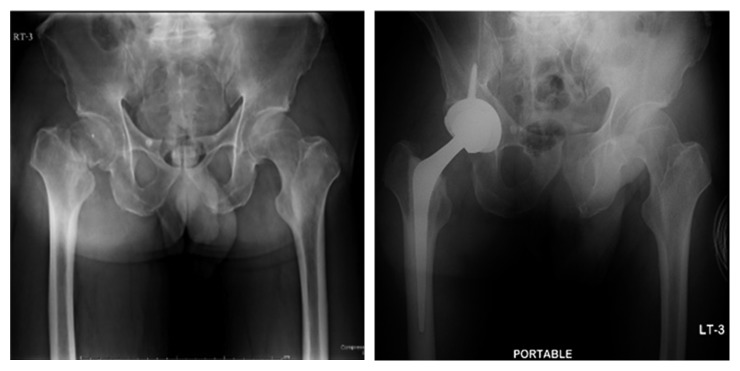
Radiograph of the hip before surgery (left) and after surgery (right).

A 57-year-old man with a history of hypertension and old left basal ganglia hemorrhage was sent to Thammasat University Hospital for right hip surgery. The patient had been well until 10 days before this evaluation, when he slipped and fell. He immediately felt a right hip pain. He was diagnosed with a fracture of the right femoral neck (Figure 1) by an orthopedist in a private hospital who subsequently referred him for an elective surgery at Thammasat University Hospital. The orthopedic team at Thammasat University Hospital planned a total hip arthroplasty for the patient. Carotid duplex ultrasound and transcranial Doppler ultrasound were evaluated before surgery without significant abnormality. Unfortunately, during surgery, the patient developed sudden hypotension and was resuscitated with 500 ml of normal saline load. Within five minutes, his blood pressure was stable.

One hour after surgery, the patient became unresponsive after weaning off anesthetic drugs. Medical consultation was requested for persistent unresponsiveness. On evaluation by the consultant physician, the patient was unable to answer any question or follow command; he had no spontaneous eye opening. The temperature was 36.8°C, the blood pressure was 110/60 mmHg, and the heart rate was 120 beats per minute. The respiratory rate was 35 breaths per minute and the oxygen saturation was between 90% and 95% while the patient was breathing via a non-rebreathing bag with 8 liters per minute of oxygen flow. The pupils were equal, round, and reactive to light with constriction from 3 mm to 2 mm. The vestibulo-ocular and gag reflexes were normal. Motor power was at grade 0/5 on the MRC scale. There was no nuchal rigidity. Deep tendon reflexes were normal. Examination of the lung revealed tachypnea and fine crepitation at both lower lung areas. Examination of the heart revealed persistent tachycardia and was otherwise normal. Urine analysis showed 2+ of occult blood, 2+ of leukocyte esterase, 1+ of protein, 10 to 20 red cells per high-power field, and 5 to 10 white cells per high-power field. Full laboratory test results before and after surgery are shown in Table 1.

**Figure 2:**
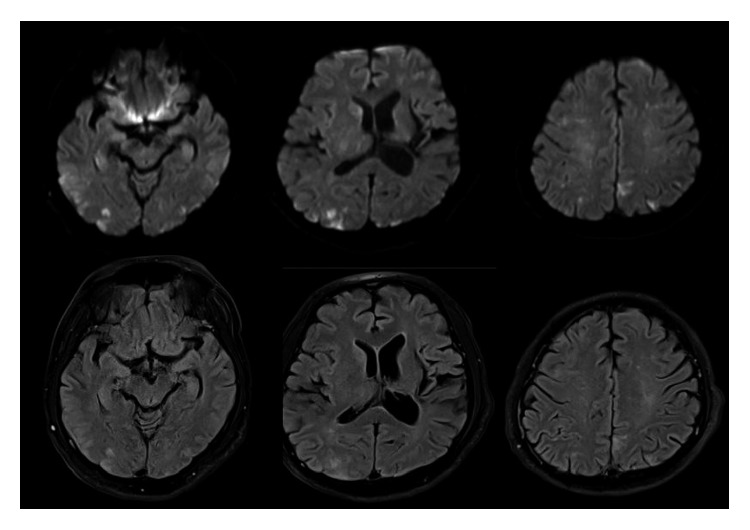
MRI brain DWI (upper) and FLAIR (lower).

The CT scans of the brain, performed without administration of intravenous contrast, revealed no evidence of acute intracranial hemorrhage, territorial infarction, or intracranial mass lesion. The MRI protocol for stroke assessment (DWI, FLAIR, T2WI, ADC, and T2^∗^), performed two hours after the CT scans of the brain, revealed multiple, scattered, small ill-defined high signal intensity lesions on T2W and FLAIR images with restricted diffusion involving the cortex and white matter of bilateral cerebral hemispheres, right caudate nucleus, and bilateral cerebellar hemispheres (Figure 2).

**Figure 3:**
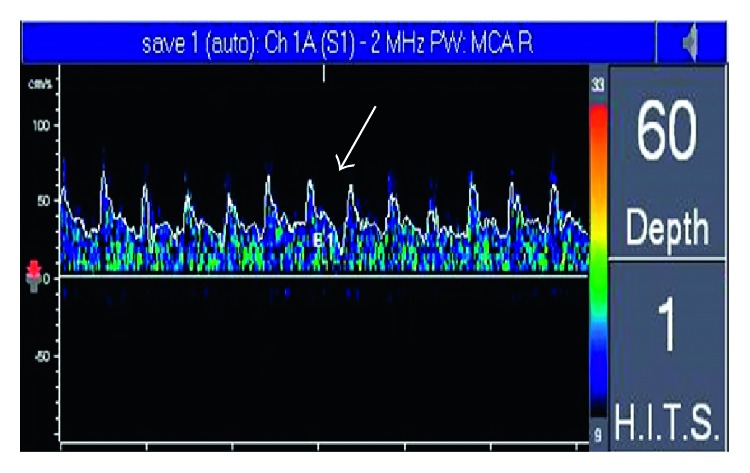
Chest radiograph at 1 hour after surgery (left) and 24 hours after surgery (right).

The chest radiograph revealed consolidation at both lungs, more prominent at both lower lung fields (Figure 3), which is consistent with acute respiratory distress syndrome. The patient was intubated and transferred to intensive care units for additional evaluation and treatment. The arterial blood gas after intubation is shown in Table 1. The CT angiogram of the chest was normal. Methylprednisolone of 500 mg intravenously once a day for 5 days was prescribed for treatment of FES and ARDS.

**Figure 4:**
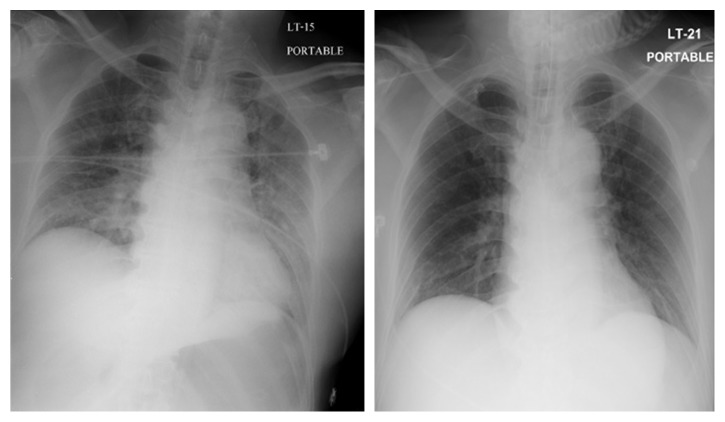
MES was detected in right MCA.

At 24 hours after the conclusion of surgery, the temperature was 37.6°C, the heart rate was 100 beats per minute, the blood pressure was 130/80 mmHg, the respiratory rate was 20 breaths per minute, and the oxygen saturation was between 95% and 100% while on the mechanical ventilator with fraction of inspired oxygen at 40%. The patient was withdrawing from painful stimulation of hands and feet. Motor power on the right side was at grade 1/5, and 2/5 on the left side on the MRC scale. The remainder of the examination was unchanged. The chest radiograph was improved (Figure 3). Transcranial Doppler ultrasound was monitored to detect microembolic signals (MES) for 30 minutes on days 1, 3, 7, and 10 after surgery. It revealed 10 high-intensity transient signals (HITS) on day 1 and 2 HITS on day 3, and they disappeared on days 7 and 10 (Figure 4). Right to left shunt was evaluated by transcranial Doppler ultrasound and echocardiogram after intravenous injection of agitated saline on days 1 and 7 after surgery. Both tests showed no abnormality.

Six days after surgery, the temperature was 37.0°C, the heart rate was 80 beats per minute, the blood pressure was 120/75 mmHg, the respiratory rate was 20 breaths per minute, and the oxygen saturation was between 95 and 100% while on mechanical the ventilator with fraction of inspired oxygen at 40%. He was somnolent but could open his eyes in response to loud verbal stimuli. Motor power on the right side was at grade 2/5 and at 3/5 on the left side on the MRC scale. Other examinations were unremarkable. He was extubated with stable oxygen saturation while breathing in ambient air. Heparin of 5000 units was administered subcutaneously for every 12 hours to prevent deep vein thrombosis. A physical therapist team was consulted to start early rehabilitation.

Fourteen days after surgery, he started to obey commands although with mild aphasia. His motor power improved to grades 3/5 and 4/5 on the right and left side, respectively, on the MRC scale. He had stable vital signs. He was subsequently discharged from the hospital after 7 days.

## 3. Discussion

FES is most commonly associated with orthopedic trauma, with highest incidence among closed, long bone fractures of the lower extremities, particularly the femur and pelvis [2]. Risk factors are male, ages 10–40 years, multiple fractures, and movement of unstable bone fractures. Possible causes of FES are shown in Table 2.

Although the pathophysiology of FES remains poorly understood, two main theories were proposed to explain pathology.

1. Mechanical theory, described by Gossling et al. [3], states that increased intramedullary pressure after an injury forces marrow to pass into injured venous sinusoids causing large fat droplets to be released into the venous system. These fat droplets then travel to the lungs and occlude pulmonary capillaries and systemic vasculatures. They can also enter the arterial circulation via a patent foramen ovale or directly through the pulmonary capillary bed, causing the characteristic neurological and dermatologic findings of FES.
2. Biochemical theory, described by Baker et al. [4], states that the clinical manifestations of FES are attributable to a proinflammatory state. Local hydrolysis of triglyceride emboli by tissue lipase produces glycerols and toxic-free fatty acids. These intermediate products lead to an injury to pneumocytes and pulmonary endothelial cells causing vasogenic and cytotoxic edema leading to a development of acute lung injury or respiratory distress syndrome. The biochemical theory helps explain the nonorthopedic forms of FES.

FES usually has an asymptomatic interval for about 12 to 72 hours after initial insult and is then followed by a classical triad of findings—respiratory insufficiency, petechial rash, and neurologic manifestation. Pulmonary manifestation is the earliest symptom and can be seen in 75% of patients [5]. Symptoms vary from dyspnea, tachypnea, and hypoxemia to ARDS. Hypoxia is the most common finding, presenting in 96% of patients. Neurological manifestation is seen in 86% of patients [6]. Symptoms are usually nonspecific, for example, headache, acute confusion, convulsion, or as severe as coma. Dermatologic manifestation is usually seen within 24 to 36 hours and usually distributed in nondependent regions of the body such as conjunctivae, head, neck, anterior thorax, or axillary areas. Most rashes typically disappear within a week. Other nonspecific symptoms include fever, thrombocytopenia, jaundice, lipuria, haematuria, and retinopathy [7]. In severe cases, FES can be complicated by disseminated intravascular coagulation, right ventricular dysfunction, shock, and death.

There are no universal criteria for diagnosis of FES. Diagnosis is made by clinical suspicion and characteristic findings on imaging methods. However, there have been three previously proposed criteria by different authors: Gurd, Schonfeld, and Lindeque (Tables 3–5). Gurd's criteria were used most widely, and the diagnosis of FES requires at least two major criteria or one major criterion plus two minor criteria. In Lindeque's criteria, FES can be diagnosed using respiratory parameters alone.

Laboratory findings in FES are usually nonspecific. Some patients may develop thrombocytopenia, anemia, or even hypofibrinogenemia. Cytological examination of the urine and sputum may show fat globules, but their diagnostic role still remains controversial. Roger et al. used bronchoalveolar lavage (BAL) to identify fat droplets in macrophages by oil red O stain. He discovered that oil red O positively stained macrophages are frequently observed in traumatic patients irrespective of the presence of FES [11].

Many imaging modalities can facilitate the diagnosis of FES, but none is specific. Chest radiographic findings may show diffuse bilateral patchy infiltrates, consistent with acute respiratory distress syndrome, although this must be differentiated from pulmonary hemorrhage or pulmonary edema. Chest films of some patients were normal. Noncontrast CT scans of the brain may be normal or reveal diffuse white matter petechial hemorrhage. MRI of the brain is sensitive for detecting FES. Its findings consist of diffuse, nonconfluent, and hyperintense lesions in both white and gray matter on diffuse wedge images and T2-weighted images, also called “star-field” pattern. These lesions gradually disappear within a few weeks to a few months [12]. However, T2-weighted MRI scans are of limited help in the hyperacute phase as these abnormalities may take several days to develop, and the findings remain highly nonspecific. Transcranial Doppler ultrasound (TCD) can also be used for detecting microembolic signals (MES) in patients with FES. Silbert et al. reviewed 14 studies that used TCD for detecting microembolic signals during orthopedic surgery. Microembolic signals were detected in all 14 studies with prevalence ranging from 20% to 100% of patients. High-intensity transient signal counts were low (<10), but high counts were present in some patients [13]. MES was detected as early as 36 hours after a long bone fracture and could persist for as long as 10 days [14].

The mainstay treatment of FES is preventive and supportive. Supportive correction of hypoxemia with supplement oxygen or mechanical ventilation is needed while the patient recovers. Additionally, monitoring of hemodynamic status and maintenance of blood volume is important in FES because shock can exacerbate the lung injury caused by FES. If patients exhibit neurologic involvement, frequent neurological examination and monitoring of intracranial pressure should be considered. Adequate anesthesia to limit sympathetic response to injury is also beneficial.

No specific drug is recommended or has strong evidence for treatment of FES. Systemic corticosteroids, which may reduce inflammation, perivascular hemorrhage, and edema, may have benefit in patients with deterioration of lung functions, but there is no systematic evidence to back up such benefit. Heparin has been proposed as a treatment of FES, due to its stimulatory effect on lipase activity and clearance of lipid from the circulation. Nevertheless, an increase in free fatty acids in the circulation could exacerbate the underlying proinflammatory physiology. Systemic anticoagulation has also been considered as a potential for FES therapy, but in the setting of trauma and pre-existing hematologic abnormalities, anticoagulants may be harmful. Early stabilization of long bone fracture is recommended to minimize bone marrow embolization into the venous system.

Prevention is the most important aspect in patients with long bone fractures. Current evidence suggests that the use of corticosteroid for prevention of FES may be considered for initial prophylaxis. A recent meta-analysis of six small randomized, controlled trials found that prophylactic corticosteroid administration reduced risk of FES development (RR 0.16, 95% CI: 0.08–0.35) and hypoxemia (RR 0.34, 95% CI 0.19–0.59) [15]. Surgical timing and techniques have important roles for preventing FES. Early surgical fixation within 24 hours after trauma has lower risk and severity than delayed fixation. External fixation or fixation with plates and screws produces less lung injury than nailing the medullary cavity, and venting the medullary canal during nailing reduces the number of emboli. Small diameter nails and unreamed nailing have been mentioned as being useful in prevention of FES [6, 16].

The prognosis of patients with FES is generally favorable. Good supportive care during patient recovery can decrease mortality rate to less than 10%. Dermatologic, neurological, and respiratory manifestation generally resolve without consequences [2].

## 4. Conclusion

The diagnosis of FES was made by clinical grounds without specific laboratory findings. However, in patients with high index of suspicion of FES, a combination of clinical criteria and MRI of the brain will enable early and accurate diagnosis of FES.

## Figures and Tables

**Table 1:** Laboratory data.

Variable	Reference range	2 days before surgery	1 hour after surgery	24 hours after surgery	48 hours after surgery
Hematocrit (%)	40–52	44.7	41	30.6	33.7
Hemoglobin (g/dl)	13.5–17.5	15.0	13.8	10.4	11.4
Red blood cell count (10^3^/*µ*l)	4.00–6.00	4.87	4.46	3.37	3.69
White blood cell count (10^3^/*µ*l)	4.0–11.0	9.7	21.6	15.1	16.1
Platelet count (10^3^/*µ*l)	140–440	353	353	234	248
Prothrombin time (sec)	11.2–13.4		14.3	15.8	
Prothrombin-time international normalized ratio			1.17	1.30	
Activated partial-thromboplastin time (sec)	22.2–29.4		28.9	29.1	
Sodium (mmol/l)	136–145	138		134	134
Potassium (mmol/l)	3.5–5.1	4.3		4.1	3.5
Chloride (mmol/l)	98–107	98		101	101
Carbon dioxide (mmol/l)	21–32	27.6		23.9	22.3
Calcium (mg/dL)	8.5–10.1			7.4	7.4
Phosphorus (mg/dL)	2.5–4.9			2.6	2.3
Magnesium (mg/dL)	1.8–2.4			2.3	2.4
Urea nitrogen (mg/dL)	7–18	15.32		19.1	21.2
Creatinine (mg/dL)	0.6–1.3	0.94		1.06	0.86
Albumin (g/dL)	3.5–5	4.1		2.5	2.3
Total protein (g/dL)	6.4–8.2	8.1		5.3	5.5
Alanine phosphatase (U/l)	12–78	52		27	64
Aspartate aminotransferase (U/l)	15–37	44		37	57
Arterial blood gas					
Fraction of inspire oxygen			0.6	0.4	
Partial pressure of CO_2_ (mmHg)	33–47		33	33	
Partial pressure of O_2_ (mmHg)	74–108		274	98	
Base excess (%)	−2 to 2		−3.1	0.80	

**Table 2:** Causes of FES.

Trauma related	Nontrauma related
1. Long bone fracture	1. Pancreatitis
2. Pelvic fracture	2. Diabetes mellitus
3. Fracture of other marrow-containing bones	3. Osteomyelitis and panniculitis
4. Orthopedic procedures	4. Bone tumor lysis
5. Soft tissue injuries (e.g., chest compression with or without rib fracture)	5. Steroid therapy
6. Burns	6. Sickle cell haemoglobinopathies
7. Liposuction	7. Alcoholic liver disease
8. Bone marrow harvesting and transplant	8. Lipid infusion
	9. Cyclosporine A solvent

The incidence of FES ranges from <1% to >30% due to a lack of universal criteria for diagnosis, and a number of mild cases of FES may be unnoticed.

**Table 3:** Guard's criteria [8] (2 major criteria or 1 major criterion plus 2 minor criteria).

Major criteria	1. Petechial rash
	2. Respiratory symptom with radiographic change
	3. Central nervous system sign unrelated to trauma or other conditions

Minor criteria	1. Tachycardia (HR 120 bpm)
	2. Pyrexia (temperature > 39°C)
	3. Retinal change (fat or petechiae)
	4. Acute thrombocytopenia
	5. Acute decrease in hemoglobin
	6. High erythrocyte sedimentation rate (ESR)
	7. Fat globules in sputum

**Table 4:** Schonfeld's criteria [9] (total score > 5 required for diagnosis).

Criteria	Point
Petechiea	5
Chest X-ray change (diffuse alveolar change)	4
Hypoxemia (PaO_2_ < 9.3 kPa)	3
Fever (temperature > 38°C)	1
Tachycardia (HR > 120 bpm)	1
Tachypnea (>30/min)	1
Confusion	1

**Table 5:** Lindeque's criteria [10].

1. Sustained PaO_2_ < 8 kPa
2. Sustained PaCO_2_ > 7.3 kPa or pH < 7.3
3. Sustained respiratory rate > 35/min despite sedation
4. Increase work of breathing, dyspnea, accessory muscle use, tachycardia, and anxiety
