# Erratum to “Fat Embolism Syndrome: A Case Report and Review Literature”

**DOI:** 10.1155/2018/3424163

**Published:** 2018-06-27

**Authors:** Nattaphol Uransilp, Sombat Muengtaweepongsa, Nuttawut Chanalithichai, Nattapol Tammachote

**Affiliations:** ^1^Fellowship in Cerebrovascular Disease and Neurovascular Ultrasound, Faculty of Medicine, Thammasat University, Pathum Thani, Thailand; ^2^Department of Internal Medicine, Faculty of Medicine, Thammasat University, Pathum Thani, Thailand; ^3^Department of Orthopedic Surgery, Faculty of Medicine, Thammasat University, Pathum Thani, Thailand

In the article titled “Fat Embolism Syndrome: A Case Report and Review Literature” [1], the legends of Figures 3 and 4 were reversed due to a production error. The correct figures and their legends are shown below.

## Figures and Tables

**Figure 3:**
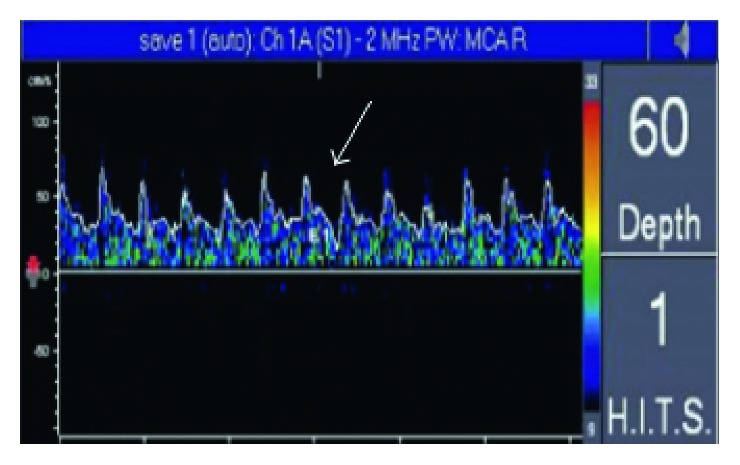
MES was detected in right MCA.

**Figure 4:**
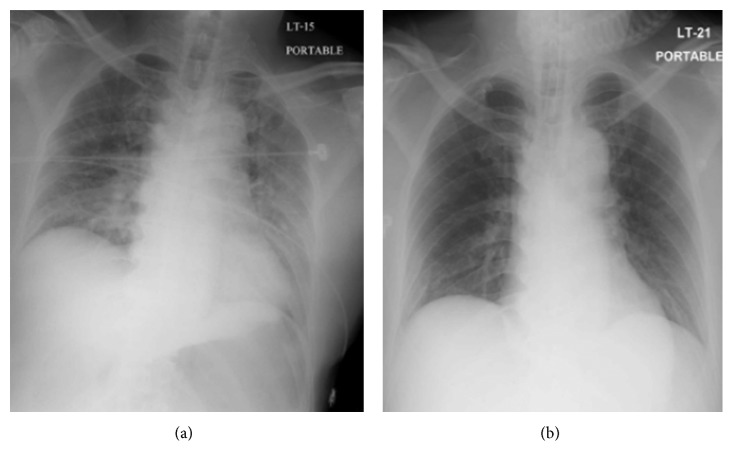
Chest radiograph at 1 hour after surgery (a) and 24 hours after surgery (b).
